# Primary squamous cell carcinoma and adenocarcinoma simultaneously occurring in the same lung lobe: a case report and literature review

**DOI:** 10.3389/fonc.2024.1402297

**Published:** 2024-05-10

**Authors:** Tianyu Zhang, Ruyuan He, Yongguang Xiao, Qing Geng

**Affiliations:** Department of Thoracic Surgery, Renmin Hospital of Wuhan University, Wuhan, China

**Keywords:** lung squamous cell carcinoma, lung adenocarcinoma, same lobe, multiple primary lung cancer, simultaneous multiple primary lung cancer

## Abstract

The co-occurrence of distinct lung cancer types within the same lobe is an exceedingly rare phenomenon. Here, we present a unique case wherein primary invasive squamous cell carcinoma and invasive adenocarcinoma concurrently manifested in the identical lung lobe. Additionally, we provide a comprehensive overview of the diagnosis and treatment approaches for multiple primary lung cancers, along with highlighting existing challenges based on the most recent guidelines. Our case underscores the importance of sampling each lesion individually, conducting separate diagnostic procedures, and determining the histological subtype for effective treatment planning irrespective of their location or size.

## Introduction

Multiple primary lung cancer (MPLC) is an infrequent form of primary lung malignancy, characterized by the simultaneous or sequential occurrence of two or more distinct lung cancers within the same individual in different regions of the lungs (1). These tumors may exhibit either similar or dissimilar histological types and are divided into simultaneous multiple primary lung cancer (sMPLC) and metachronous multiple primary lung cancer (mMPLC) based on the diagnosis interval exceeding 6 months (2). It has been reported that the incidence of MPLC in lung cancer is about 0.7% to 15%, and the incidence of sMPLC in MPLC in Chinese population is about 0.3% to 1.2% (3). In recent years the utilization of multislice spiral computed tomography (CT) and positron emission tomography (PET) scanning has significantly enhanced early detection rates for MPLC, prompting increased attention to this condition. Nevertheless, sMPLC and mMPLC are still relatively rare, especially within the same lobe, and most of MPLC are of the same histological type. Adenocarcinoma represents the predominant histological subtype observed in patients with MPLC (80%-99%), followed by squamous cell carcinoma (13%) (4). We report a rare case of sMPLC, characterized by simultaneous occurrence of two lung cancers with distinct histologic types within the same lobe. In this study, we aim to to summarize the latest advances in the diagnosis and treatment of MPLC based on the latest guidelines, and discuss the potential prognostic impact of two different histological types of cancer in the same lobe, in order to provide valuable insights for clinical practice.

## Case report

**Figure 1:**
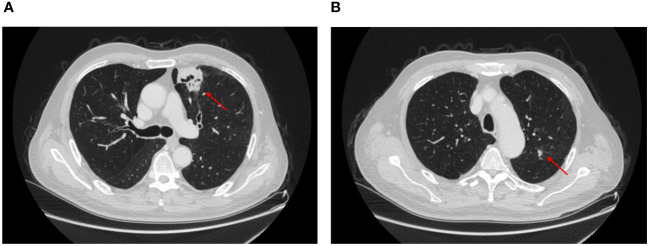
CT showed the location of the pulmonary nodule in the left upper lung. **(A)** squamous cell carcinoma, **(B)** adenocarcinoma.

A 65-year-old man was admitted to the hospital with intermittent hemoptysis lasting for 8 months. 8 months ago, he first developed blood-streaky sputum, and then 4 months ago his symptoms began to worsen significantly with hemoptysis, and underwent a high resolution CT at the local hospital, which revealed a nodule on the left upper lung of 2.2*2.6 cm. CT-guided aspiration biopsy was performed and pathology showed that there was fibrotic proliferation of the lung tissues, with widened alveolar septa, and a large number of lymphocytic infiltrates and lymphoid follicle deposits in the alveolar lumen. Hemoptysis improved after hemostatic and anti-infectious treatment. In the last two weeks, the hemoptysis aggravated, and the high-resolution CT in our hospital suggested that the left upper lung had a cavitary lung mass of 3.9*3cm, and a nodule of 1cm in the left upper lobe near the interlobar fissure (**Figure 1**), which was treated with symptomatic anti-infective and hemostasis treatment without significant improvement. Fiberoptic bronchoscopy reveals hemorrhage in the opening of the upper lobe of the left lung and it was recommended that re-excision biopsy and PET-CT again, but was forcefully refused, and he was given cerebral contrast enhanced magnetic resonance scan, thoracic and abdominal CT with i.v. contrast, cervical lymph nodes ultrasonography and radionuclide bone scan. There were no suspicious metastatic lesions. After MDT consultation and discussion with oncologists and pulmonary physicians, the patient underwent lobectomy of the left upper lung and mediastinal lymph node systematic clearance under single-port thoracoscopy (wedge resection was performed first, and then lobectomy of the lung was carried out after the frozen section pathology suggested squamous cell carcinoma during the operation).

**Figure 2:**
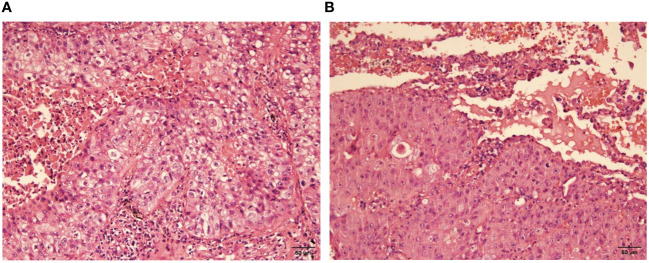
Moderately-poorly differentiated squamous cell carcinoma (H-E stain, x400), nonkeratinized. **(A)** the view of cancerous fields, **(B)** the view of both normal and cancerous fields.

**Figure 3:**
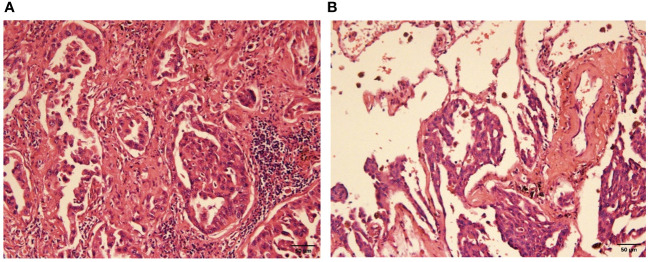
Poorly differentiated adenocarcinoma (H-E stain, x200). **(A)** the view of cancerous fields, **(B)** the view of both normal and cancerous fields.

Pathological examination of the left upper lobe revealed two lesions. Postoperative pathology suggests non-keratinizing invasive squamous cell carcinoma of moderate to poor differentiation without evidence of vascular or nerve invasion (**Figure 2**). Pleural invasion grade was categorized as PL1. Immunohistochemical findings demonstrated positivity for CK5/6 (+), P40 (+), P63 (+), Ki-67 (approximately 50% in hot spots), while Napsin A (-) and TTF-1 (-). Special staining results indicated presence of elastic fibers. The second tumor (anatomically superior) was revealed invasive adenocarcinoma. The proportions of tumor growth patterns of the papillary, acinar, micropapillary, cribriform parts were 40%, 30%, 20%, 10% respectively (**Figure 3**). The maximum diameter of invasion measured at 1.2cm, exhibiting poor differentiation and vascular invasion; STAS was poorly positive without any observed invasion of visceral pleura or nerves, lymph node (Group 5, 6, 7, 9, 10, 11, 12) without metastasis. Immunohistochemical results indicated CK7 (+), Ki-67 (+) at approximately 20%, Napsin A (+), TTF-1 (+), P53 weakly positive as wild type, and P40 (-).

The patient’s final diagnosis was lung squamous carcinoma combined with adenocarcinoma, T3N0M0, stage IIB according to the ninth edition of the tumor node metastasis (TNM) staging system and successfully discharged from the hospital after five days, and was proposed to receive adjuvant treatment after one month postoperatively.

## Discussion

Currently, the clinical diagnosis of MPLC is predominantly based on the Martini-Melamed diagnostic criteria (1), which primarily rely on histologic features, tumor location, presence or absence of carcinoma in situ, lymphatic invasion, metastasis, and other empirical characteristics. The American College of Chest Physicians (ACCP) has also emphasized the necessity for distinct molecular genetic characteristics among different lesions and recommended extending the tumor-free interval for mMPLC to 4 years (5, 6). The 2023 NCCN guidelines underscore that diagnosing MPLC necessitates multidisciplinary treatment involving thoracic surgery, pathology, radiology, and oncology while streamlining the diagnostic process to aid clinicians in making optimal medical decisions (7).

Accurate diagnosis of MPLC is essential. Various methods have been proposed to help the diagnosis, including DNA ploidy patterns and p53 gene mutation homology assays (8). However, clinical manifestations, imaging examination and pathological features are still the main factors for the comprehensive diagnosis of MPLC (9). Next-generation sequencing has emerged as a promising tool for precise diagnosis and prognostic assessment in lung cancer compared to traditional histopathological evaluation (10). Although EGFR and KRAS mutations can help to diagnose the synchronous occurrence of primary adenocarcinoma, combined immunohistochemistry may be a better choice due to intratumor heterogeneity limitations (11). Unfortunately, based on the patient’s wishes, only immunohistochemistry was performed in this case, and sequencing and other related examinations were not completed. However, we suggest that if the relevant conditions are met, further studies such as sequencing are necessary for accurate diagnosis and prognosis evaluation.

Accurate identification of MPLC is crucial for selecting appropriate treatment strategies. Patients with MPLC are at risk of receiving inappropriate treatment based solely on imaging and histopathological staging reports (12). In the past, multiple pulmonary nodules were often misinterpreted as metastatic lesions, leading to a lack of surgical intervention. Once diagnosed with MPLC, surgery should be promptly recommended if there are no clinical contraindications; subsequent radiotherapy or image-guided thermal ablation may follow suit. If local therapy is not feasible for the patient, palliative care or observation should be considered. Studies have demonstrated that surgical interventions can lead to favorable prognoses in patients with MPLC (13–15). Although the diagnostic criteria for MPLC have been established, there is still a lack of unified authoritative principles and guidelines for surgical treatment. Furthermore, microwave ablation, targeted therapy, immunotherapy and other technologies are increasingly utilized in patients with MPLC (16, 17). However, due to the multi-driver gene mutation characteristics of MPLC and the diversity of immune microenvironment, more prospective randomized trials are necessary to validate their efficacy. Additionally, positive driver genes-targeted therapy and immunotherapy have demonstrated promising outcomes in postoperative adjuvant phase (18, 19). The high frequency of gene mutations in MPLCs particularly EGFR mutations provides objective evidence for the effectiveness of targeted therapy (20). Nevertheless, differences in mutation between different lesions within the same patient (4) emphasize the importance of evaluating each lesion’s mutation status before considering targeted therapy for MPLC patients. Moreover, it is noteworthy that lymph node metastasis is an independent prognostic factor for MPLC; however, its characteristics and patterns remain unclear as there are limited relevant studies with differing perspectives. Given the future emergence of lung cancer screening programs and the rising incidence of MPLC cases, it is crucial to develop unified diagnostic criteria and treatment plans while standardizing post-treatment monitoring for optimal management.

In summary, MPLC exhibits distinct characteristics in imaging, pathology, and molecular genetics, necessitating multidisciplinary collaboration for the development of diagnosis and treatment strategies. Surgical intervention currently remains the primary therapeutic approach for MPLC patients with solitary lung lesions, offering a favorable prognosis when performed at an early stage (21).

Furthermore, despite the recent increase in the incidence of MPLC, there have been limited reports documenting synchronous adenocarcinoma and squamous cell carcinoma occurring in the same lobe (22–24),which are very old. Similar to our case, these reported patients were predominantly male smokers aged between 55 and 70 years. The cases of lung cancer with various histologic types that have been reported in recent years are summarized in **Table 1**. It remains unknown whether there are other undiagnosed and unreported cases, as well as the potential impact of gender, lifestyle factors, and other variables on this type of case. We speculate that the true incidence of MPLC may be higher than what is currently detected by CT and PET (28). Moreover, since the onset of the COVID-19 pandemic in 2019, there has been a significant rise in MPLC incidence due to increased utilization of CT scans among patients (29); however, no association between COVID-19 and MPLC has been reported. What’s more, most of the sMPLC reported at present are in the early stage, and the prognosis of surgery is good. The effect of related treatments such as chemotherapy and immunotherapy for patients with advanced sMPLC remains to be studied. The patient in this case was still in the early stage of the disease, and the prognosis of the operation was good. The two lesions recovered well after operation. We suggest that for patients with sMPLC with different histopathological features, after excluding intrapulmonary metastasis, multiple lesions should be treated as independent individuals for postoperative treatment and long-term follow-up.

**Table 1:** The reported lung cancer cases with different histopathological types in recent years.

Year of Report	Patients	Histopathological Types	Definitive Diagnosis	Citation
2017	78-year-old man	small cell lung cancer; adenocarcinoma; squamous cell carcinoma	cSCLC	(25)
2019	82-year-old man	Squamous Cell Cancer(left lower superior segment); Small Cell Lung Cancer(left upper lobe)	sMPLC	(3)
2021	69-year-old man	colloid adenocarcinoma(right lower lobe); squamous cell carcinoma(right upper lobe)	sDPLC	(26)
2022	67-year-old woman	adenoid cystic carcinoma(the opening of the left main bronchus); adenocarcinoma (the right lower lobe)	MPLC	(27)

In conclusion, the rarity of this case highlights the importance of obtaining separate diagnostic samples from each lesion in patients with sMPLC, irrespective of tumor location and size, to determine individual T, N, and M stages for each lesion. Furthermore, a comprehensive targeted treatment plan should be developed to enhance patient survival and improve their quality of life. It is crucial to emphasize that resectability should not be excluded for different lesions within the same lobe and they should be treated individually regardless of lesion location. Additionally, MPLC requires increased attention along with long-term follow-up; therefore, it is imperative to establish standardized guidelines for MPLC treatment at the earliest opportunity. With continuous advancements in molecular biomarkers and genetic analysis techniques, rapid and accurate diagnosis of MPLC as well as evaluation of treatment options will no longer pose significant challenges.

## Data availability statement

The original contributions presented in the study are included in the article/supplementary material. Further inquiries can be directed to the corresponding authors.

## Ethics statement

Written informed consent was obtained from the individual(s) for the publication of any potentially identifiable images or data included in this article. Written informed consent was obtained from the participant/patient(s) for the publication of this case report.

## Author contributions

TZ: Writing – original draft. RH: Writing – original draft. YX: Writing – review & editing. QG: Writing – review & editing.
